# *Tryonia*, a new taenitidoid fern genus segregated from *Jamesonia* and *Eriosorus* (Pteridaceae)

**DOI:** 10.3897/phytokeys.35.6886

**Published:** 2014-02-26

**Authors:** Alyssa T. Cochran, Jefferson Prado, Eric Schuettpelz

**Affiliations:** 1 Department of Biology and Marine Biology, University of North Carolina Wilmington, 601 South College Road, Wilmington, NC 28403-5915, U.S.A.; 2 Instituto de Botânica, C.P. 68041, 04045-972, São Paulo, SP, Brazil; 3 Department of Botany (MRC 166), National Museum of Natural History, Smithsonian Institution, PO Box 37012, Washington DC 20013-7012, U.S.A.

**Keywords:** Brazil, phylogeny, pteridophytes, Taenitidoideae, taxonomy

## Abstract

The Neotropical fern genera *Eriosorus* and *Jamesonia* have long been thought of as close relatives. Molecular phylogenetic studies have confirmed this notion but have also revealed that neither genus is monophyletic with respect to the other. As a result, all known species of *Eriosorus* were recently subsumed under the older generic name *Jamesonia*. Here, through an analysis of a four-gene plastid dataset, we show that several species traditionally treated in *Eriosorus* are in fact more closely related to other taenitidoid fern genera (namely *Austrogramme*, *Pterozonium*, *Syngramma*, and *Taenitis*) than they are to the large *Jamesonia* *sensu lato* clade. *Tryonia* Schuettp., J.Prado & A.T.Cochran **gen. nov.** is described to accommodate these species and four new combinations are provided. *Tryonia* is confined to southeastern Brazil and adjacent Uruguay; it is distinct (from most species of *Jamesonia*) in having stramineous rachises.

## Introduction

**Figure 1.**
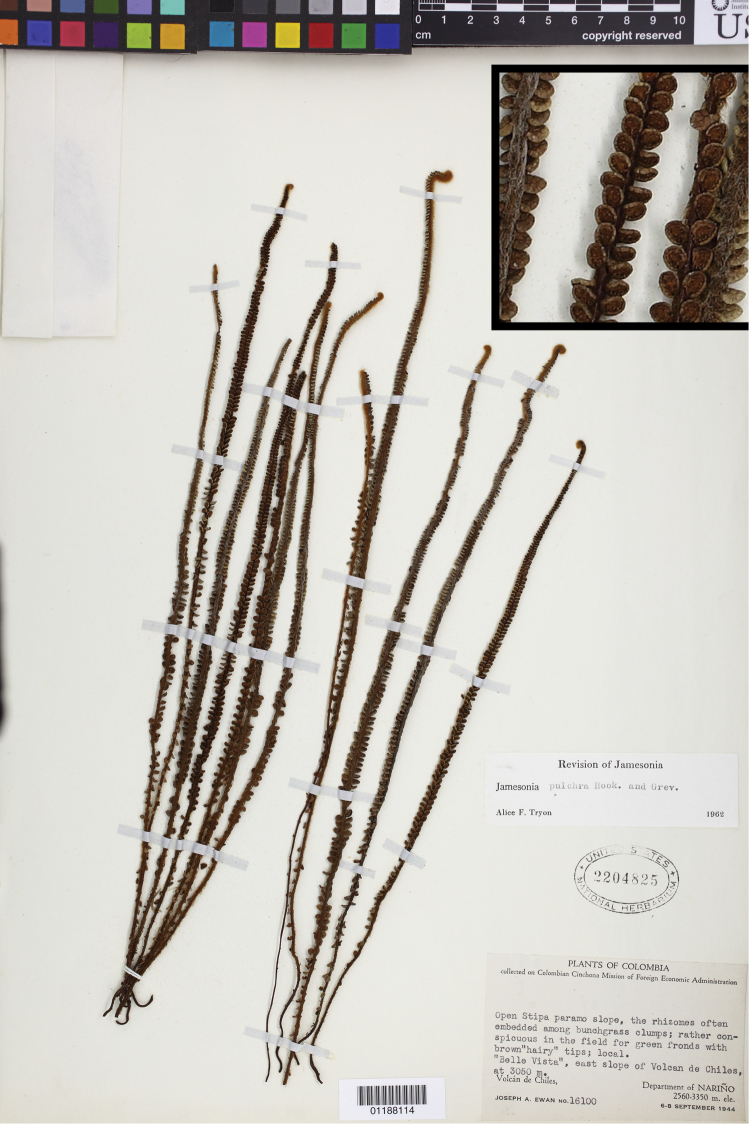
*Jamesonia pulchra* Hook. & Grev., the type species of *Jamesonia*. Ewan 16100 (US), inset detail of (castaneous) rachis magnified 4×.

**Figure 2.**
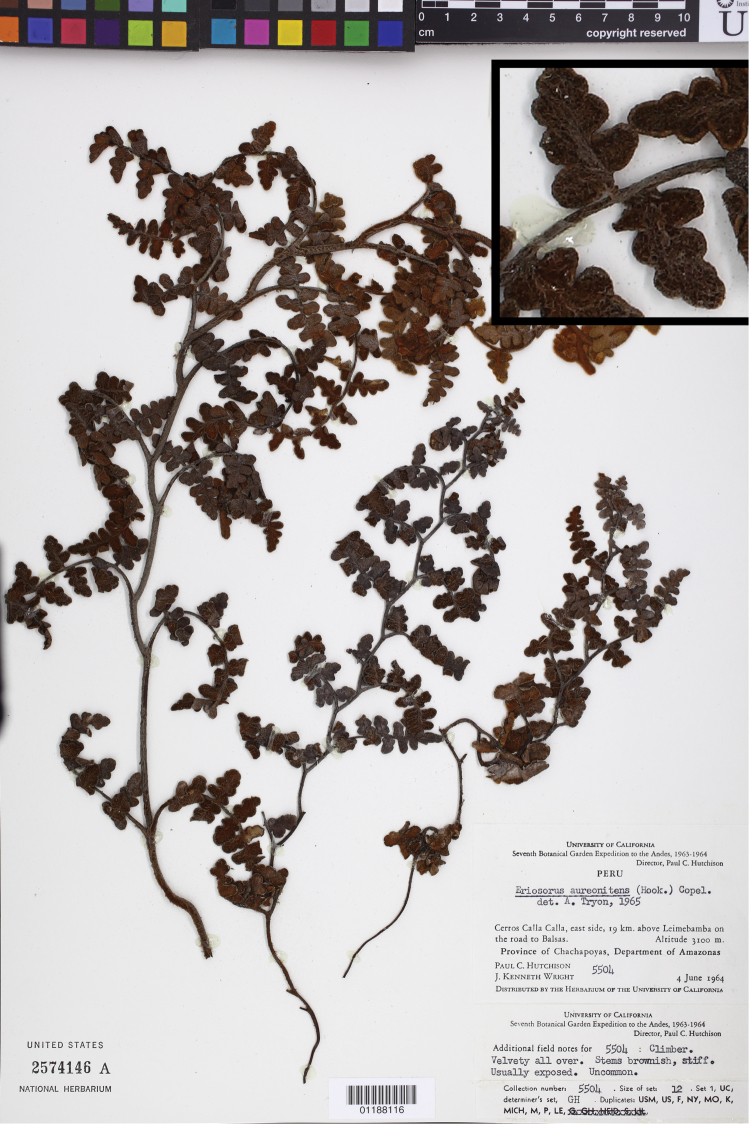
*Jamesonia aureonitens* (Hook.) Christenh., the type species of *Eriosorus*. Hutchison 5504 (US), inset detail of (castaneous) rachis magnified 4×.

**Figure 3.**
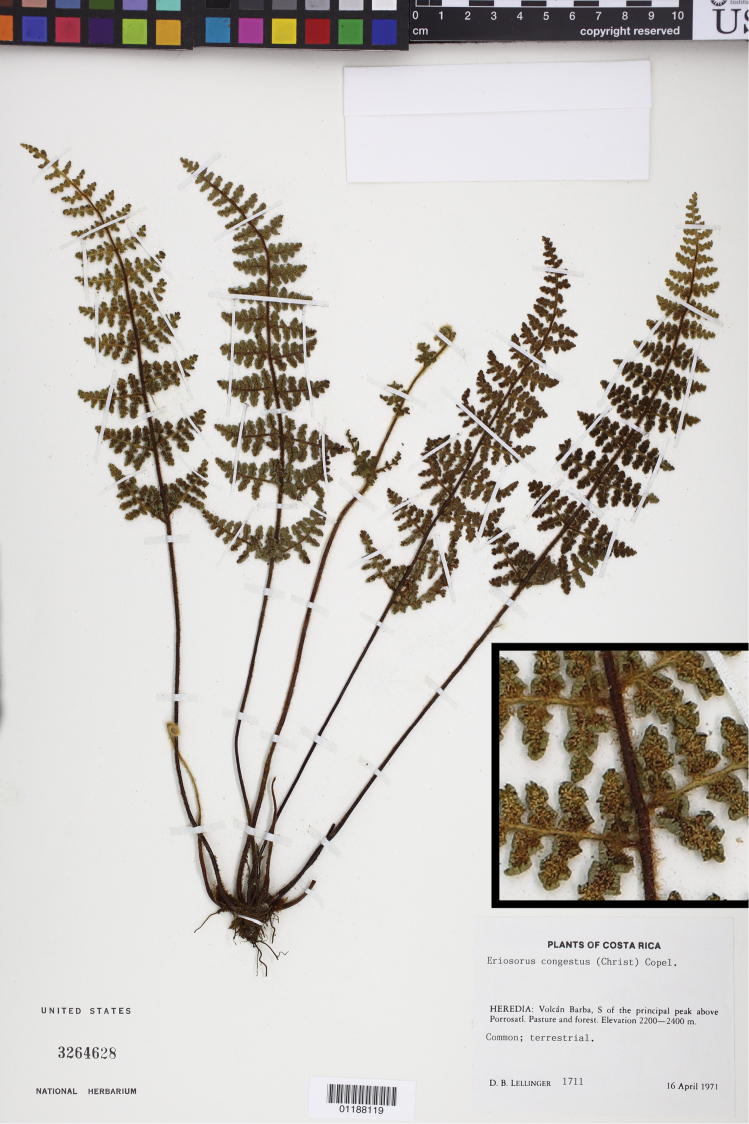
*Jamesonia congesta* (Christ) Christenh., a species with generalized morphology (Tryon 1970) previously classified in *Eriosorus*. Lellinger 1711 (US), inset detail of (castaneous) rachis magnified 4×.

The Neotropical genus *Jamesonia* Hook. & Grev. *sensu stricto* is among the most distinctive of all fern genera. It has linear, indeterminate leaves bearing highly reduced, coriaceous pinnae covered with dense pubescence (Tryon 1962; Fig. 1). These morphological characteristics are generally considered to be an adaptation to the high-elevation Andean páramo habitats where most *Jamesonia* species reside (Tryon et al. 1990). Based on reproductive and other cryptic morphological characteristics, *Jamesonia* has long been thought to be closely related to the genus *Eriosorus* Fée (Tryon 1962, 1970, Tryon and Tryon 1982). *Eriosorus* mostly occupies middle-elevation habitats in the Andes and its leaves are much more typical of ferns, usually being very dissected and rather delicate in texture (Tryon 1970; Figs 2, 3). Recent analyses have demonstrated that *Jamesonia* is both nested within *Eriosorus* and polyphyletic (Prado et al. 2007, Sánchez-Baracaldo 2004a, 2004b, Schneider et al. 2013, Schuettpelz et al. 2007), supporting the hypothesis of Tryon (1962, 1970) that the unique morphology of *Jamesonia* evolved independently multiple times. This finding prompted the recent recombination of all known species of *Eriosorus* into *Jamesonia* (*sensu lato*, Christenhusz et al. 2011).

**Figure 4.**
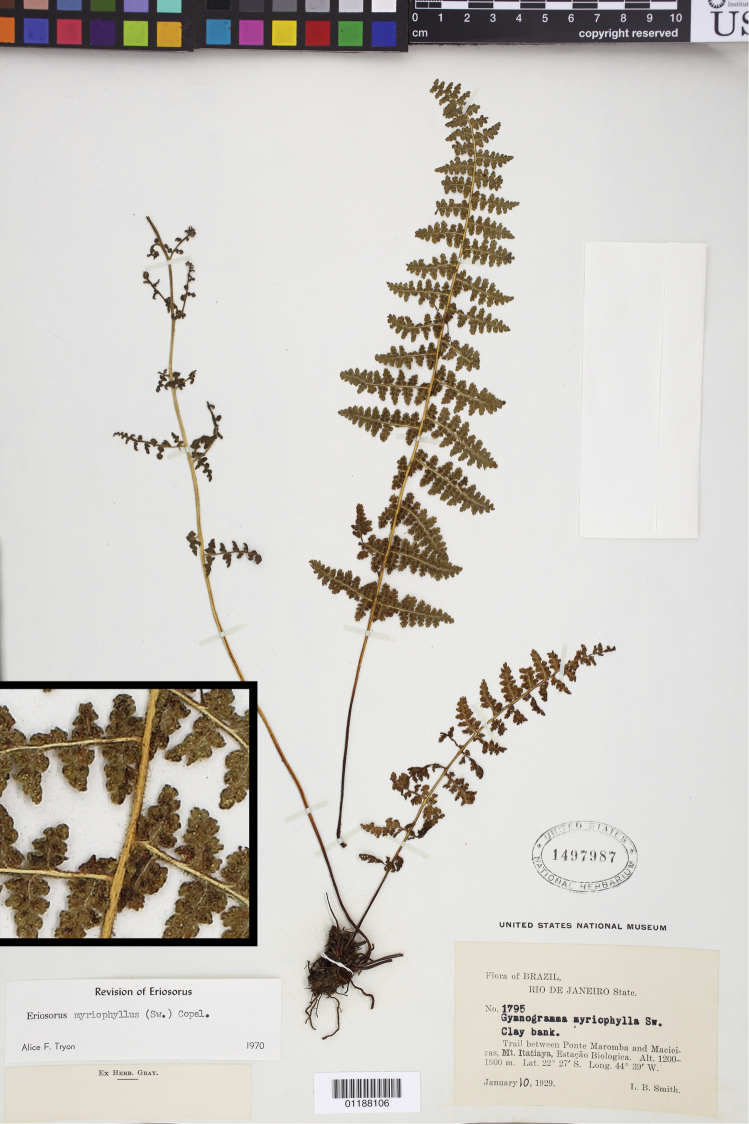
*Tryonia myriophylla* (Sw.) Schuettp., J.Prado & A.T.Cochran, the type species of *Tryonia*. Smith 1795 (US), inset detail of (stramineous) rachis magnified 4×.

Although it is clear that species of *Jamesonia* *sensu stricto* are intermixed with those previously assigned to *Eriosorus*, relationships remain rather poorly supported and additional studies are needed to better resolve the evolutionary history of this group. With that said, the isolated phylogenetic position revealed for one Brazilian species requires special attention. In the most comprehensive study of *Jamesonia* *sensu lato* to date (Sánchez-Baracaldo 2004b), two accessions of *Eriosorus myriophyllus* (Sw.) Copel. (Fig. 4) were resolved together and well supported as sister to the remainder of *Jamesonia* *sensu lato.* However, it is clear from the phylogram included in the Sánchez-Baracaldo (2004b) study that these accessions are genetically more similar to the outgroup used than they are to the remainder of the ingroup, suggesting that the phylogenetic position of *Eriosorus myriophyllus* may be an artifact of including a single outgroup genus (*Pterozonium* Fée). Subsequent analyses with a broader phylogenetic context but including fewer exemplars from within *Jamesonia* *sensu lato*, actually found *Eriosorus myriophyllus* to be most closely related to the genus *Taenitis* Willd. ex Schkuhr (Prado et al. 2007, Schneider et al. 2013).

Here, through analyses of a four-gene (*atpA*, *chlL*, *rbcL*, and *rps4*) plastid dataset that incorporates many *Eriosorus* and *Jamesonia* *sensu stricto* species, as well as a broad sampling of related genera, we aim to better resolve the phylogenetic position of *Eriosorus myriophyllus* and allied species. Based on our results, we describe a new genus, *Tryonia* Schuettp., J.Prado & A.T.Cochran, to accommodate this species and its closest allies.

## Methods

### Sampling

A total of thirty-eight collections were sampled for the phylogenetic analysis, including four individuals of *Eriosorus myriophyllus*, nine other species of *Eriosorus*, eight *Jamesonia* *sensu stricto* species, and seventeen additional species representing other genera in the taenitidoid clade (Prado et al. 2007, Sánchez-Baracaldo 2004a, Schuettpelz et al. 2007, Table 1).

**Table 1.** Collections included in our phylogenetic analyses supporting the recognition of *Tryonia*, with voucher information and corresponding GenBank accession numbers.

Species	Voucher	*atpA*	*chlL*	*rbcL*	*rps4*	FLDB^†^
*Actiniopteris dimorpha* Pic.Serm.	Schneider s.n. (GOET)	EF452066	KJ416295	EF452130	KJ416352	3515
*Actiniopteris semiflabellata* Pic.Serm.	Smith s.n. (UC)	KJ416270	KJ416296	KJ416326	KJ416353	3742
*Anogramma leptophylla* (L.) Link	Schuettpelz 1079 (DUKE)	KJ416271	KJ416297	KJ416327	KJ416354	4822
*Austrogramme decipiens* (Mett.) Hennipman	van der Werff 16114 (UC)	NA	NA	NA	AF321702	NA
*Austrogramme marginata* (Mett.) E.Fourn.	Hodel 1454 (UC)	NA	NA	NA	AY357704	NA
*Cosentinia vellea* (Aiton) Tod.	Larsson 55 (UPS)	KJ416272	KJ416298	KJ416328	KJ416355	8670
*Jamesonia alstonii* A.F.Tryon	Moran 8248 (DUKE)	KJ416273	KJ416299	KJ416329	KJ416356	5587
*Jamesonia blepharum* A.F.Tryon	Schuettpelz 269 (DUKE)	KJ416274	KJ416300	EF452154	KJ416357	2437
*Jamesonia brasiliensis* Christ	Schuettpelz 1444 (SP)	KJ416275	KJ416301	KJ416330	KJ416358	8379
*Jamesonia cheilanthoides* (Sw.) Christenh.	Rothfels 3964 (DUKE)	KJ416276	KJ416302	KJ416331	KJ416359	7694
*Jamesonia congesta* (Christ) Christenh.	Grusz 08-036 (DUKE)	KJ416277	KJ416303	KJ416332	KJ416360	5272
*Jamesonia elongata* (Grev. & Hook.) J.Sm.	Rothfels 3602 (DUKE)	KJ416278	KJ416304	KJ416333	KJ416361	7362
*Jamesonia flexuosa* (Kunth) Christenh.	Rothfels 08-042 (DUKE)	KJ416279	KJ416305	KJ416334	KJ416362	5273
*Jamesonia goudotii* (Hieron.) C.Chr.	Rothfels 3694 (DUKE)	KJ416280	KJ416306	KJ416335	KJ416363	7414
*Jamesonia hirta* (Kunth) Christenh.	Rothfels 3669 (DUKE)	KJ416281	KJ416307	KJ416336	KJ416364	7397
*Jamesonia insignis* (Kuhn) Christenh.	Salino 3010 (UC)	NA	NA	NA	AF321708	NA
*Jamesonia pulchra* Hook. & Grev.	Sánchez-Baracaldo 306 (UC)	NA	NA	NA	AF321746	NA
*Jamesonia rotundifolia* Fée	Sundue 1357 (DUKE)	KJ416282	KJ416308	KJ416337	KJ416365	6049
*Jamesonia scammaniae* A.F.Tryon	Rothfels 2631 (DUKE)	KJ416283	KJ416309	KJ416338	KJ416366	5588
*Jamesonia verticalis* Kunze	Rothfels 3638 (DUKE)	KJ416284	KJ416310	KJ416339	KJ416367	7386
*Jamesonia warscewiczii* (Mett.) Christenh.	Grusz 08-039 (DUKE)	KJ416285	KJ416311	KJ416340	KJ416368	5275
*Onychium japonicum* (Thunb.) Kunze	Schneider s.n. (GOET)	EF452107	KJ416312	KJ416341	NA	3463
*Onychium lucidum* (D.Don) Spreng.	Schuettpelz 1161 (DUKE)	KJ416286	KJ416313	KJ416342	NA	4904
*Pityrogramma austroamericana* Domin	Schuettpelz 301 (DUKE)	EF452112	KJ416314	EF452166	KJ416369	2561
*Pityrogramma chaerophylla* (Desv.) Domin	Prado 2178 (SP)	KJ416287	KJ416315	KJ416343	KJ416370	8755
*Pityrogramma jamesonii* (Baker) Domin	Moran 7592 (NY)	EF463857	KJ416316	EF452167	KJ416371	3769
*Pterozonium brevifrons* (A.C.Sm.) Lellinger	Schuettpelz 285 (DUKE)	EF452124	KJ416317	EF452175	KJ416372	2453
*Pterozonium cyclosorum* A.C.Sm.	Brewer 1006 (UC)	NA	NA	NA	AF321703	NA
*Pterozonium reniforme* (Mart.) Fée	Brewer 1005 (UC)	NA	NA	NA	AF321704	NA
*Syngramma quinata* (Hook.) Carr.	Kessler 2273 (L)	NA	NA	NA	AF321701	NA
*Taenitis blechnoides* (Willd.) Sw.	Schuettpelz 689 (DUKE)	KJ416288	KJ416318	KJ416344	KJ416373	4102
*Taenitis interrupta* Hook. & Grev.	Schuettpelz 851 (DUKE)	KJ416289	KJ416319	KJ416345	KJ416374	4270
*Tryonia areniticola* (Schwartsb. & Labiak) Schuettp., J.Prado & A.T.Cochran	Prado 2169 (SP)	NA	KJ416320	KJ416346	KJ416375	8433
*Tryonia myriophylla* (Sw.) Schuettp., J.Prado & A.T.Cochran	Schuettpelz 1411 (SP)	KJ416290	KJ416321	KJ416347	KJ416376	8345
*Tryonia myriophylla* (Sw.) Schuettp., J.Prado & A.T.Cochran	Schuettpelz 1449 (SP)	KJ416291	KJ416322	KJ416348	KJ416377	8384
*Tryonia myriophylla* (Sw.) Schuettp., J.Prado & A.T.Cochran	Schuettpelz 1461 (SP)	KJ416292	KJ416323	KJ416349	KJ416378	8396
*Tryonia myriophylla* (Sw.) Schuettp., J.Prado & A.T.Cochran	Prado 2186 (SP)	KJ416293	KJ416324	KJ416350	NA	8753
*Tryonia schwackeana* (Christ) Schuettp., J.Prado & A.T.Cochran	Schuettpelz 1433 (SP)	KJ416294	KJ416325	KJ416351	KJ416379	8367

^†^ Fern Lab Database voucher number (see http://fernlab.biology.duke.edu for additional information concerning these collections)

### DNA extraction, amplification, and sequencing

Genomic DNA was typically extracted using a modified CTAB protocol (Doyle and Doyle 1987), as described in detail in Beck et al. (2011). Four plastid gene regions (*atpA*, *chlL*, *rbcL*, and *rps4*) were amplified using the polymerase chain reaction (PCR). Each reaction incorporated 13.6 µl ultrapure water, 2 µl buffer (10×), 2 µl dNTPs (2 mM each), 0.2 µl Choice-Taq DNA Polymerase (5 units/µl, Denville Scientific), 0.2 µl BSA (10 mg/ml), 1 µl forward primer (10 µM), 1 µl reverse primer (10 µM), and 1 µl template DNA (primer details are provided for each gene in Table 2). All thermal cycling protocols employed an initial denaturation step (95 °C for 2 min), 35 amplification cycles, and a final elongation step (71 °C for 5 min). Each amplification cycle involved a denaturation step (95 °C for 0.5 min), an annealing step (50 °C for 0.5 min for *atpA*, *chlL*, and *rps4*; 45 °C for 0.5 min for *rbcL*), and an elongation step (71 °C for 1 min for *atpA* and *chlL*; 71 °C for 1.5 min for *rps4* and *rbcL*).

**Table 2.** Primers utilized in this study supporting the recognition of *Tryonia*.

Region	Name	Type	Sequence	Reference
*atpA*	atpA-F1	Forward	GAATCTGATAATGTTGGGGCTG	This study
*atpA*	atpA-R1	Reverse	AAACATCTCCNGGATAYGCTTC	This study
*chlL*	chlL-F1	Forward	GRATTGGMAARTCAACAACTAGCTG	This study
*chlL*	chlL-R1	Reverse	CBAGTACRGGCATGGGRCAAGCTTC	This study
*rbcL*	ES-rbcL-1F	Forward	ATGTCACCACAAACGGAGACTAAAGC	Schuettpelz and Pryer 2007
*rbcL*	ES-rbcL-1361R	Reverse	TCAGGACTCCACTTACTAGCTTCACG	Schuettpelz and Pryer 2007
*rbcL*	ES-rbcL-628F	Forward^†^	CCATTYATGCGTTGGAGAGATCG	Schuettpelz and Pryer 2007
*rbcL*	ES-rbcL-654R	Reverse^†^	GAARCGATCTCTCCAACGCAT	Schuettpelz and Pryer 2007
*rps4*	rps5	Forward	ATGTCCCGTTATCGAGGACCT	Souza-Chies et al. 1997
*rps4*	trnS	Reverse	TACCGAGGGTTCGAATC	Souza-Chies et al. 1997

^†^ Primer used only for sequencing.

Amplifications were visualized using standard gel electrophoresis and imaging approaches. Unincorporated nucleotides and primers were removed from successful reactions by adding 1.0 µl Shrimp Alkaline Phosphatase (1 unit/µl) and 0.5 µl Exonuclease I (10 units/µl) to each reaction and incubating at 37 °C for 15 min. Reactions were then heated to 80 °C for 15 min to inactivate the enzymes.

Sequencing reactions were carried out, in both directions, with the amplification primers, following a standard protocol (Schuettpelz and Pryer 2007). For *rbcL*, two additional (internal) sequencing primers were utilized (Table 2). Sequencing reactions were cleaned using the ZR-96 DNA Sequencing Clean-up Kit (Zymo Research), according to the manufacturer’s protocol. Sealed plates were submitted to Operon (Huntsville, Alabama) for sequencing.

Sequencing reads were independently (for each PCR product) assembled and edited using Sequencher (Gene Codes Corporation). The 110 new consensus sequences were added to the Fern Lab Database (http://fernlab.biology.duke.edu) and deposited into GenBank (Table 1). For four (of thirty-eight) collections, we could only obtain three of the four gene regions targeted (Table 1). For six collections, an *atpA* and/or *rbcL* sequence had already been published; these existing sequences (from Schuettpelz and Pryer 2007 and Schuettpelz et al. 2007) were obtained directly from GenBank, as were seven *rps4* sequences (from Sánchez-Baracaldo 2004a, 2004b) corresponding to species not otherwise available to us (Table 1). All new and existing sequences were aligned, by gene region, using Mesquite (Maddison and Maddison 2011). The final *atpA*, *chlL*, *rbcL*, and *rps4* datasets included 30, 31, 31, and 35 taxa, respectively (see Table 3 for additional details concerning our alignments).

**Table 3.** Details for the alignments analyzed in this study supporting the recognition of *Tryonia*.

		Characters			Data	Bipartitions
Dataset	Taxa	Total	Included	Variable	Missing^†^	Supported^‡^
*atpA*	30	1506	629	113	1.04%	11
*chlL*	31	523	523	120	0.92%	15
*rbcL*	31	1309	1309	250	0.39%	15
*rps4*	35	1176	560	177	1.77%	17
Combined	38	4514	3021	660	17.76%	25

^†^ Calculation based on included characters

^‡^ Bayesian posterior probability ≥ 0.95

### Phylogenetic analyses

Bayesian phylogenetic analyses were conducted independently for each of the four single-gene datasets using MRBAYES version 3.2.1 (Huelsenbeck and Ronquist 2001, Ronquist and Huelsenbeck 2003). These Bayesian analyses utilized the GTR+Γ+I model of sequence evolution (the most complex model available) and consisted of four independent runs per dataset, each utilizing four chains and proceeding for five million generations, with trees sampled every 4000 generations. After completion of each analysis, we examined the standard deviation of split frequencies among the runs, plotted the output parameter estimates using Tracer 1.5 (Rambaut and Drummond 2009), and very conservatively excluded the first 250 trees (one million generations) from each run. A majority-rule consensus phylogeny with clade posterior probabilities was then calculated from the remaining 4000 trees, for each gene. Based on earlier studies with broader sampling (Prado et al. 2007, Sánchez-Baracaldo 2004a), we rooted our resulting gene trees with *Actiniopteris* and *Onychium*.

We compared the results of our single-gene analyses, looking for conflicts that were supported by a Bayesian posterior probability ≥ 0.95. Finding none, we concatenated the four datasets. The resulting 38-taxon combined dataset was analyzed as above, but with model parameters estimated and optimized separately for each gene and each run proceeding for 20 million generations. We sampled trees every 16,000 generations and excluded the first four million generations from each run prior to calculating a majority-rule consensus phylogeny with clade posterior probabilities.

## Results

The four single-gene (*atpA*, *chlL*, *rbcL*, and *rps4*) datasets contained varying amounts of phylogenetic signal, providing significant support (Bayesian posterior probability, BPP ≥ 0.95) for as few as 11 and as many as 17 bipartitions (Table 3). The single-gene trees were largely consistent in their resolved relationships (trees not shown) and there were no well-supported (BPP ≥ 0.95) conflicts among them.

**Figure 5.**
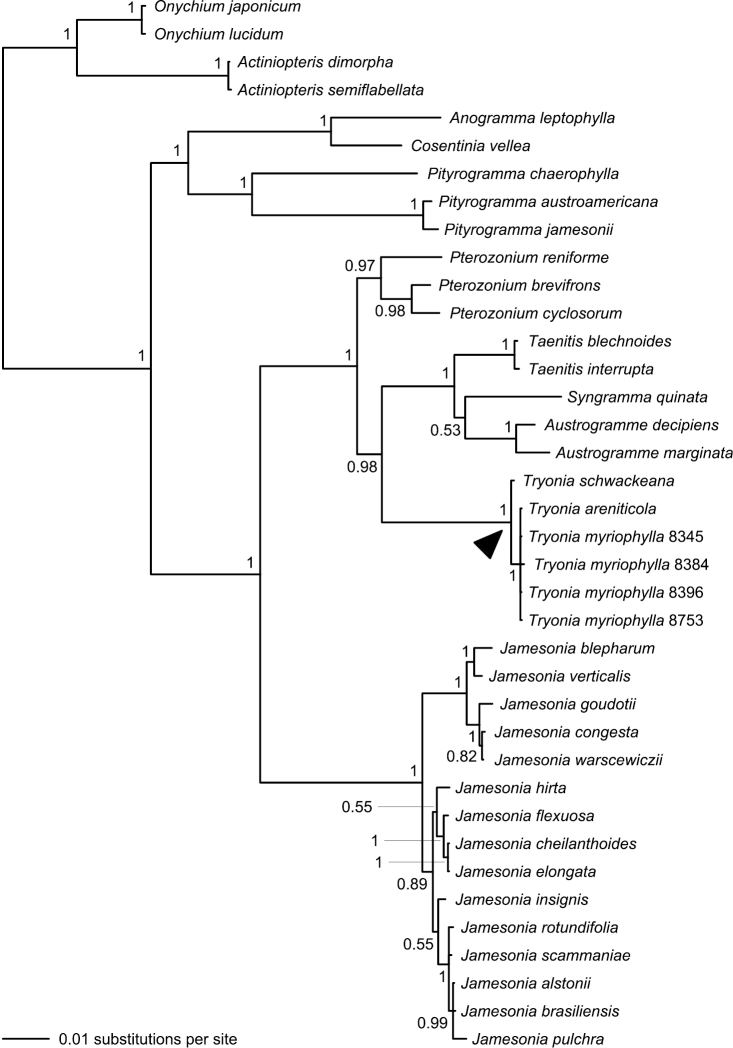
Phylogeny resulting from Bayesian analysis of our combined four-gene (*atpA*, *chlL*, *rbcL*, and *rps4*) plastid dataset. Posterior probabilities (≥ 0.50) are provided at the nodes. Note that species now treated in *Tryonia* (black arrowhead) are distinct from *Jamesonia*, the genus in which these species were most recently placed. Numbers provided for *Tryonia myriophylla* samples are Fern Lab Database voucher numbers (Table 1).

Our combined four-gene dataset comprised a total of 4514 characters, of which 660 were variable (Table 3). Analysis of this dataset resulted in a phylogeny with considerably improved support relative to the single-gene phylogenies; 25 bipartitions had a BPP ≥ 0.95 (Fig. 5). The separation of *Actiniopteris* and *Onychium* from the remaining taenitidoid genera was well supported (BPP = 1.00). *Anogramma*, *Cosentinia*, and *Pityrogramma* formed a well-supported clade that was, in turn, well-supported as sister to a robust clade including *Austrogramme*, *Pterozonium*, *Syngramma*, *Taenitis*, and all sampled species previously assigned to either *Jamesonia* or *Eriosorus* (Fig. 5).

The vast majority of our *Jamesonia* *sensu lato* collections come together in a clade on a rather long branch; within this clade branches are short and support is frequently lacking. Six samples previously included within *Jamesonia* *sensu lato* are not allied to that larger clade, but rather are embedded within a well-supported clade that also contains *Austrogramme*, *Pterozonium*, *Syngramma*, and *Taenitis* (Fig. 5).

## Discussion

Most species previously assigned to *Eriosorus* and *Jamesonia* *sensu stricto* have been consistently resolved together in a well-supported clade (Prado et al. 2007, Sánchez-Baracaldo 2004a, 2004b, Schneider et al. 2013, Schuettpelz et al. 2007). And, although support for relationships within this large clade has been generally lacking, the hypothesis that *Jamesonia* *sensu stricto* was derived from within *Eriosorus* (Tryon 1962, 1970) has received considerable backing. In our combined analysis, we too find strong support for a clade containing most sampled *Eriosorus* and *Jamesonia* *sensu stricto* species (Fig. 5). Additionally, we find strong support for some of its constituent internal nodes, which indicate that neither *Eriosorus* nor *Jamesonia* *sensu stricto* is monophyletic. Phylogenetic analyses incorporating a more comprehensive sample of taxa and a greater number of markers will ultimately be necessary to fully understand evolutionary relationships within this clade. However, based solely on the evidence to date, it is abundantly clear that *Jamesonia* and *Eriosorus* (as typically circumscribed) cannot both be recognized, assuming monophyly as a criterion for generic delimitation. With *Jamesonia* being the older name (published in 1830, versus 1852 for *Eriosorus*), the recombination of all known species of *Eriosorus* into *Jamesonia* in Christenhusz et al. (2011) was mostly warranted.

*Eriosorus myriophyllus* was shown by Prado et al. (2007), Sánchez-Baracaldo (2004b), and Schneider et al. (2013) to be isolated relative to most other species previously assigned to *Eriosorus* or *Jamesonia* *sensu stricto.* Here, we find *Eriosorus myriophyllus* and two previously unsampled species of *Eriosorus* to be more closely related to *Austrogramme*, *Pterozonium*, *Syngramma*, and *Taenitis* than to *Jamesonia* (as newly circumscribed herein, Fig. 5). Support for this relationship is strong (BPP = 1.00) and the implications are significant if monophyly is used as a criterion for generic delimitation. Because the type of *Jamesonia* (*Jamesonia pulchra* Hook. & Grev.) is resolved well within the large *Jamesonia* clade and the type of *Eriosorus* (*Eriosorus aureonitens* (Hook.) Copel.) shows clear morphological and geographical affinities to this clade, and because there are no other generic names available for the *Eriosorus myriophyllus* group, we here describe a new genus—*Tryonia* (see below)—to accommodate the isolated species.

In her monograph of *Eriosorus*, Tryon (1970) identified several small groups of closely allied species. Among these was the species pair of *Eriosorus myriophyllus* and *Eriosorus sellowianus* (with *Eriosorus schwackeanus* considered by her to be a synonym of*Eriosorus sellowianus*). This group corresponds perfectly to our proposed circumscription of *Tryonia*. We find *Eriosorus myriophyllus*, *Eriosorus schwackeanus* (which we consider to be distinct from *Eriosorus sellowianus*), and the recently described *Eriosorus areniticola* (Schwartsburd and Labiak 2008) to form a genetically isolated clade of closely related species (Fig. 5). New combinations for these species, along with the unsampled *Eriosorus sellowianus*, are provided below.

Based on our current dataset, we do not consider the precise phylogenetic position of *Tryonia* (within the *Austrogramme*, *Pterozonium*, *Syngramma*, *Taenitis*, and *Tryonia* clade) to be fully resolved. Although our combined analysis clearly places *Tryonia* sister to *Austrogramme*, *Syngramma*, and *Taenitis* (collectively), this relationship is not well supported in any single-gene analysis. The *atpA* and *rbcL* datasets do place *Tryonia* sister to *Taenitis* (*atpA* and *rbcL* sequences were not available for *Austrogramme* and *Syngramma*), but support is lacking (BPP = 0.61 and 0.83, respectively). Likewise, the *rps4* dataset resolves *Tryonia* as sister to *Austrogramme*, *Syngramma*, and *Taenitis* without significant support (BPP = 0.88). Strong single-gene support for the precise position of *Tryonia* only comes from the *chlL* dataset, where *Tryonia* is most closely related to *Pterozonium* (BPP = 1.00).

Two of the species of *Tryonia* included in our phylogenetic analysis (*Tryonia areniticola* and *Tryonia schwackeana*) are endemic to Brazil; the third sampled species (*Tryonia myriophylla*) also occurs in Uruguay, near its border with the Brazilian state of Rio Grande do Sul. Although the Andes are the center of diversity for *Jamesonia* (as newly circumscribed herein), this genus is not entirely geographically distinct from *Tryonia*. In the recently published Catálogo de Plantas e Fungos do Brasil, a total of nine species are ascribed to *Eriosorus* or *Jamesonia* (Prado 2010). Only three of these species noted for Brazil (*Eriosorus areniticola*, *Eriosorus myriophyllus*, and *Eriosorus schwackeanus*) are resolved as sister to *Austrogramme*, *Syngramma*, and *Taenitis*. We found *Eriosorus cheilanthoides*, *Eriosorus insignis*, and *Jamesonia brasiliensis* to be embedded within the *Jamesonia* clade (Fig. 5) and *Eriosorus rufescens* was resolved within *Jamesonia* in an earlier study (Sánchez-Baracaldo 2004b). As for the remaining Brazilian species that have yet to be included in a phylogenetic study, one (*Eriosorus sellowianus*) shows clear morphological affinities to, and is here considered to be a member of, *Tryonia*; the other (*Eriosorus biardii*) appears, based on morphology, to be best accommodated in *Jamesonia*. Regardless of the ultimate phylogenetic placement of these two unsampled species, the genus *Tryonia* can be described as wholly endemic to Brazil and Uruguay.

## Taxonomy

### Tryonia

Schuettp., J.Prado & A.T.Cochran gen. nov.

urn:lsid:ipni.org:names:77136217-1

http://species-id.net/wiki/Tryonia

**Figure 6.**
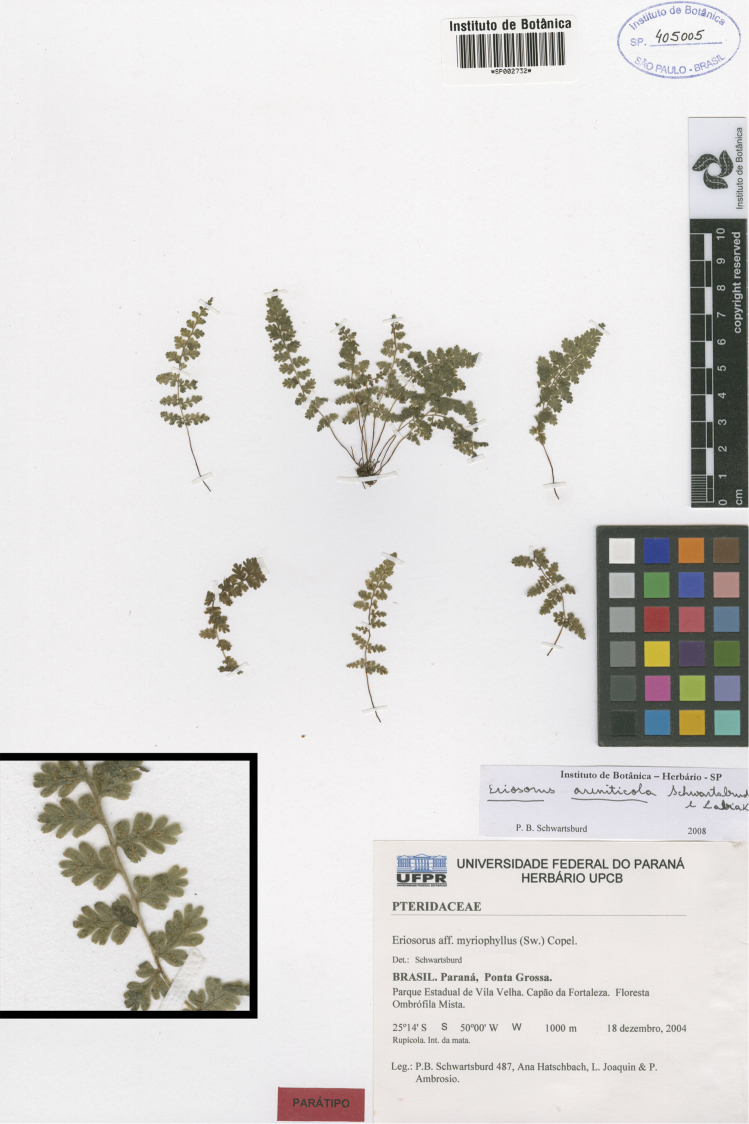
*Tryonia areniticola* (Schwartsb. & Labiak) Schuettp., J.Prado & A.T.Cochran. Schwartsburd 487 (SP), inset detail of (stramineous) rachis magnified 4×.

Figs 4 , 6 [Fig F7] [Fig F8] –9

#### Diagnosis

*Similar to some species of* Jamesonia, *but with stramineous rather than castaneous rachises*.

#### Type.

*Tryonia myriophylla* (Sw.) Schuettp., J.Prado & A.T.Cochran, comb. nov., *Gymnogramma myriophylla* Sw., Kongl. Vetensk. Acad. Handl. 1817(1): 58. 1817.

#### Description.

Plants terrestrial, rupicolous, or saxicolous. Rhizomes creeping to erect at apex, compact, with appressed hairs or crispate bristles, sometimes rigid, ruddy brown, darker at the base. Fronds erect, 6–100 cm long; petioles terete or sulcate adaxially, brown at base and stramineous distally, from 1/8 as long to equal the length of the lamina, densely to sparsely pubescent, the hairs short and erect or long and crispate, hyaline or reddish brown at the cell junctions, glandular or non-glandular; laminae linear to elongate-triangular, 1 or 2-pinnate-pinnatissect to 1–3-pinnate-pinnatifid, 4.0–48 cm long, 1.0–14 cm wide, determinate; rachises straight, sometimes slightly flexuous, terete or sulcate adaxially, stramineous, pubescent, the hairs like those of the petioles; pinnae ascending to patent to the rachis, oblong to deltate, 0.5–10 cm long, 0.5–5 cm wide, membranaceous to herbaceous, densely to sparsely pubescent on both surfaces, the hairs glandular, hyaline or with the terminal cell light to dark reddish brown, 2–5-celled, or hairs non-glandular, hyaline or reddish brown at the cell junctions, 2–5(–7)-celled; ultimate segments entire and round or emarginate; veins free. Sporangia borne along the veins, short-stalked, stalks 1–2-celled, stomia with 2–4 indurated cells; spores trilete, tetrahedral-globose, with an equatorial flange, distal face coarsely tuberculate, proximal face with prominent ridges, brown, 40–60 µm (Fig. 9).

**Figure 7. F7:**
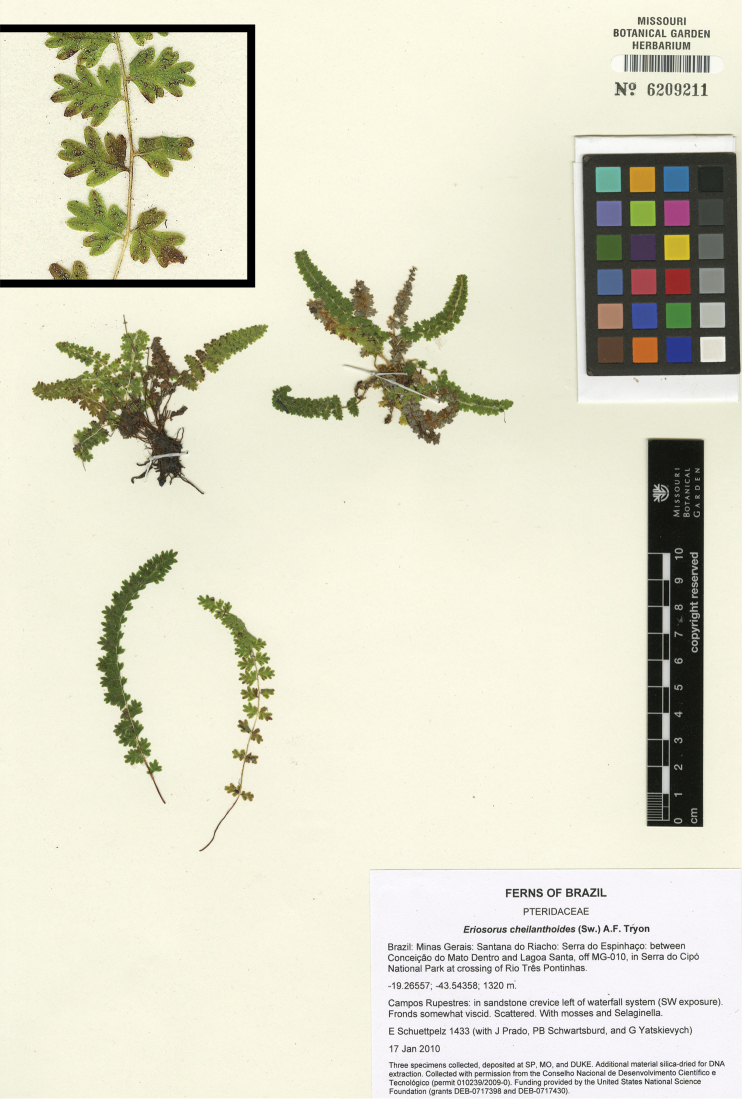
*Tryonia schwackeana* (Christ) Schuettp., J.Prado & A.T.Cochran. Schuettpelz 1433 (MO), inset detail of (stramineous) rachis magnified 4×. Image modified from http://www.tropicos.org/Image/100140486.

**Figure 8. F8:**
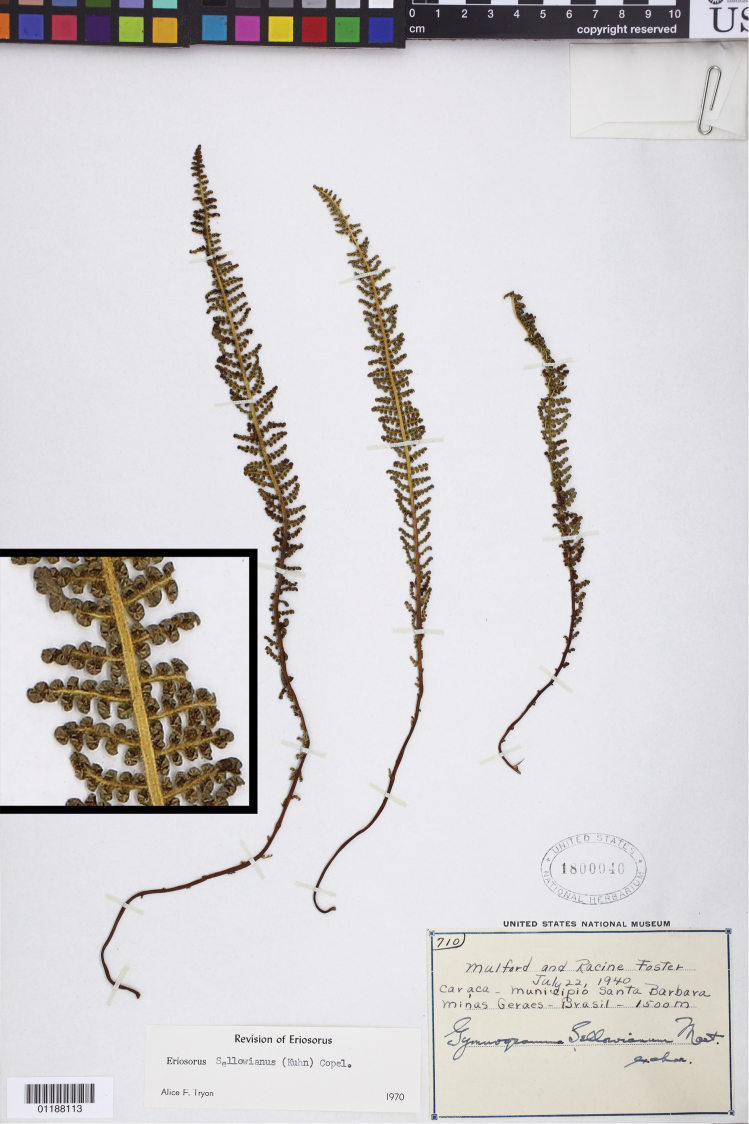
*Tryonia sellowiana* (Kuhn) Schuettp., J.Prado & A.T.Cochran. Mulford 710 (US), inset detail of (stramineous) rachis magnified 4×.

**Figure 9.**
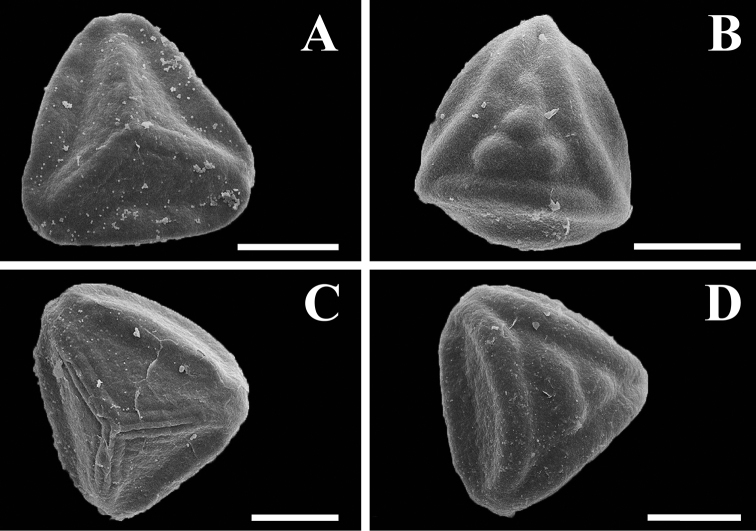
Spores of *Tryonia*. A. *Tryonia myriophylla* proximal view, Wacket s.n. (US) **B** *Tryonia myriophylla* distal view, Wacket s.n. (US) **C** *Tryonia areniticola* proximal view, Kummrow 2773 (US) **D** *Tryonia areniticola* distal view, Kummrow 2773 (US). All scale bars are 20 µm.

#### Etymology.

The generic name honors Dr. Alice Faber Tryon, who made extraordinary contributions to fern systematics and published taxonomic revisions of both *Jamesonia* *sensu stricto* and *Eriosorus* (from which *Tryonia* is segregated herein).

#### Distribution.

*Tryonia* occurs primarily in southeastern Brazil. However, one species (*Tryonia myriophylla*) can also be found in Uruguay (Cerro Largo: Sierra Souza), near the Brazilian border. The genus is mostly restricted to the Atlantic Forest, along shaded streams, on damp shaded sandstone, or in more open places (but here shaded by shrubs); 600–2300 m.

#### Discussion.

*Tryonia* can be distinguished most readily from *Jamesonia* by its stramineous rachises, but its gross morphology is also reasonably distinct. Tryon (1970) referred to the leaves of *Tryonia myriophylla* as “generalized” (i.e., elongate-triangular and well developed). She drew a distinction between them and the “specialized” (i.e., either complex and scandent or compact and linear) leaves of *Jamesonia* *sensu stricto* and many other species at the time placed in *Eriosorus*, as well as between them and the “intermediate” (i.e., falling between the two extremes) leaves of other species she treated in *Eriosorus*. Although the Andean *Jamesonia congesta* also has “generalized” leaves, it is readily distinguished from *Tryonia* by its rachis color. The only species of *Jamesonia* with occasionally stramineous rachises (*Jamesonia flexuosa*) has “specialized” (complex and scandent) leaves. Spores of *Tryonia* (Fig. 9) and *Jamesonia* are basically indistinguishable.

*Tryonia* comprises the following species.

### Tryonia areniticola

(Schwartsb. & Labiak) Schuettp., J.Prado & A.T.Cochran comb. nov.

urn:lsid:ipni.org:names:77136218-1

http://species-id.net/wiki/Tryonia_areniticola

Figs 6 , 9

Jamesonia areniticola Synonym: (Schwartsb. & Labiak) Christenh. (Phytotaxa 19: 20. 2011).

#### Basionym.

*Eriosorus areniticola* Schwartsb. & Labiak (Amer. Fern J. 98: 160. 2008).

#### Type.

**Brazil:** Paraná: Jaguariaíva: Parque Estadual do Cerrado, 12 April 1994, *P.H. Labiak 182* (holotype: UPCB; isotypes: SP!, UC).

#### Distribution.

Brazil: Paraná, Rio Grande do Sul, Santa Catarina (probably), and São Paulo.

#### Discussion.

Based on the gene regions included in our analysis, we found *Tryonia areniticola* to be genetically indistinguishable from *Tryonia myriophylla*, despite the presence of several morphological differences (Schwartsburd and Labiak 2008). Further studies that include nuclear markers will be necessary.

### Tryonia myriophylla

(Sw.) Schuettp., J.Prado & A.T.Cochran comb. nov.

urn:lsid:ipni.org:names:77136219-1

http://species-id.net/wiki/Tryonia_myriophylla

Figs 4 , 9

Psilogramme myriophylla Synonyms: (Sw.) Kuhn (Festschr. 50 Jähr. Jub. Königstädt. Realschule Berlin 339. 1882); *Eriosorus myriophyllus* (Sw.) Copel. (Gen. Fil. 58. 1947); *Jamesonia myriophylla* (Sw.) Christenh. (Phytotaxa 19: 21. 2011).

#### Basionym.

*Gymnogramma myriophylla* Sw. (Kongl. Vetensk. Acad. Handl. 1817(1): 58. 1817).

#### Type.

**Brazil:** [Minas Gerais]: Villa Rica [now Ouro Preto], Aug 1815, *G.W. Freyriss* *s.n*. (lectotype [designated by Tryon, 1970]: S-R-2467, image!; isolectotypes: BM 000936677, image!, S-R-2469, image!).

#### Distribution.

Brazil: Bahia, Espírito Santo, Minas Gerais, Paraná, Rio de Janeiro, Santa Catarina, São Paulo, and Rio Grande do Sul. Uruguay: Cerro Largo.

### Tryonia schwackeana

(Christ) Schuettp., J.Prado & A.T.Cochran comb. nov.

urn:lsid:ipni.org:names:77136220-1

http://species-id.net/wiki/Tryonia_schwackeana

Fig. 7

Eriosorus schwackeanus Synonym: (Christ) Copel. (Gen. Fil. 59. 1947).

#### Basionym.

*Gymnogramma schwackeana* Christ in Schwacke (Pl. Nov. Mineiras 2.18. 1900).

#### Type.

**Brazil:** [Minas Gerais]: Ouro Preto, *C.A.W. Schwacke 9389* (lectotype [designated by Tryon, 1970]: P 00603566, image!; isolectotype: GH 00021287, image!).

#### Distribution.

Brazil: Bahia and Minas Gerais.

### Tryonia sellowiana

(Kuhn) Schuettp., J.Prado & A.T.Cochran comb. nov.

urn:lsid:ipni.org:names:77136221-1

http://species-id.net/wiki/Tryonia_sellowiana

Fig. 8

Psilogramme sellowiana Synonyms: (Mett. ex Kuhn) Kuhn (Festschr. 50 Jähr. Jub. Königstädt. Realschule Berlin 339. 1882); *Eriosorus sellowianus* (Mett. ex Kuhn) Copel. (Gen. Fil. 59. 1947); *Jamesonia sellowiana* (Mett. ex Kuhn) Christenh. (Phytotaxa 19: 21. 2011).

#### Basionym.

*Gymnogramma sellowiana* Mett. ex Kuhn (Linnaea 36:69. 1869).

#### Type.

**Brazil**, *Sello 1365* (lectotype [designated by Tryon, 1970]: B-Herb. Mett., image!; isolectotype: B, image!)

#### Distribution.

Brazil: Minas Gerais.

## Supplementary Material

XML Treatment for Tryonia

XML Treatment for Tryonia areniticola

XML Treatment for Tryonia myriophylla

XML Treatment for Tryonia schwackeana

XML Treatment for Tryonia sellowiana
